# Comparison between bone alkaline phosphatase immunoassay and electrophoresis technique in hemodialysis patients

**DOI:** 10.2478/jomb-2019-0048

**Published:** 2020-01-23

**Authors:** Neda Milinković, Marija Sarić-Matutinović, Svetlana Pejanović, Svetlana Ignjatović

**Affiliations:** 1 University of Belgrade, Faculty of Pharmacy, Department of Medical Biochemistry, Belgrade; 2 Clinical Center of Serbia, Clinic of Nephrology, Belgrade

**Keywords:** bone alkaline phosphatase, electrophoresis, hemodialysis, immunoassay, imunoodređivanje, hemodijaliza, elektroforeza, koštana alkalna fosfataza

## Abstract

**Background:**

Problem of the variability between the different methods using for bone alkaline phosphatase (bALP) determination greately influences the clinical significance of bALP as direct marker of bone metabolism. The aim of this study was to compare immunoassay with electrophoresis technique for bALP determination.

**Methods:**

We measured bALP in 71 patients on hemo - dialysis with agar gel electrophoresis (ISO-PAL, SEBIA) and immunoassay (OSTASE, Beckman Coulter).

**Results:**

The analyzed methods showed significant correlation (Spearman's rho: 0.776, P < 0.01), but we found statistically significant (P < 0.01) positive bias (27%) for the results measured by immunoassay. In support of this, using electrophoresis technique we have detected presence of the intestinal isoenzymes of alkaline phosphatase in 55% of patients with median value of 30% of the total alkaline phoshatase and presence of liver-2 alkaline phosphatase isoform in 42% of patients with median value of 16.6%. The Kendall's W of 0.787 (P<0.0001) revealed significant concordance between two analysed methods. Cusum test showed no significant deviation from linearity (P=0.850).

**Conclusions:**

Despite good agreement between immuno - assay methods and electrophoresis technique for bALP determination, interchangeability between these two methods is questionable. Although immunoassays are increasingly used, as fully automated methods, in a large number of laboratories and become routine methods for bALP determination, it should be beared in mind, besides various interferences, also the heterogeneity of the bALP itself, especially in patients on hemodialysis.

## Abstract (sr)

**Uvod:**

Problem varijabilnosti između različitih metoda pomoću kojih se određuje koštani izoezim alkalne fosfataze (bALP) u velikoj meri utiče na klinički značaj bALP kao direktnog markera metabolizma kostiju. Cilj ove studije bio je poređenje imunohemijskog testa sa elektroforetskom tehnikom za određivanje bALP.

**Metode:**

bALP je određen kod 71 pacijenta na hemodijalizi, agar gel elektroforetskom tehnikom (ISO-PAL, SEBIA) i imunohemijskom metodom (OSTASE, Beckman Coulter).

**Rezultati:**

Analizirane metode pokazale su značajno slaganje (Spearmanov rho: 0,776, P < 0,01), ali je uočen statistički značajan (P < 0,01) pozitivan bias (27%) za rezultate izmerene imunohemijskom metodom. U prilog ovome, primenom elektroforetske tehnike, otkrili smo prisustvo intestinalnih izoenzima alkalne fosfataze u 55% bolesnika, sa srednjom vrednosti od 30% ukupne alkalne fostataze, kao i prisustvo jetrenih izoformi alkalne fosfataze kod 42% bolesnika sa srednjom vrednosti od 16,6%. Kendall-ov W od 0,787 (P < 0,0001) ukazivao je na značajno slaganje između dve analizirane metode. Cusumov test nije pokazao značajno odstupanje od linearnosti (P = 0,850).

**Zaključak:**

Uprkos tome što postoji dobro slaganje između imunohemijske metode i elektroforetske tehnike za određivanje bALP-a, međusobna zamenjivost ove dve metode je pod znakom pitanja. Iako se imunohemijske metode sve više koriste kao potpuno automatizovane, u velikom broju laboratorija i postaju rutinske metode za određivanje bALP-a, treba imati na umu, osim različitih interferencija, i heterogenost samog bALP, posebno kod bolesnika na hemodijalizi.

## Introduction

Bone alkaline phosphatase isoenzyme (bALP) is considered a useful biomarker for assessing the level of bone metabolism in patients on dialysis [1]. In spite of the fact that it directly depicts the bone formation cells metabolism and is present in the guidelines as a recommended biomarker, it has not yet been fully implemented in practice. The basic problem is that there is no harmonization of the methods used to determine this isoenzyme, and there is no agreement between the recommended cut-off values in the estimation of bone metabolism levels. Various studies that examined the relationship between biomarkers and bone histology in patients on hemodialysis (HD) have not agreed in proper bALP cut-off value for difference between low, normal and high bone turnover [2] [3]. In addition to the different methodology used, the heterogeneity of histological abnormalities in HD patients is also a problem [3]. Originally used electrophoretic methods were not easily feasible and practically applicable as a routine in most laboratories. The development of automation and application of immunochemical methods in routine practice has made it easier to determine bALP. However, immunochemical determination is subject to numerous interferences, different specificity of the antibodies used for detection, and method standardization problems [4].

It is considered that the associated determination of bALP and parathyroid hormone (PTH) does not increase the diagnostic capability of PTH as a recommended biomarker to distinguish between low, normal and high bone turnover [3]. Also, the literature data indicate that there is a discrepancy between the levels of bALP and PTH, although in most cases bALP determination correlates with PTH in estimating the level of bone metabolism [5] [6] [7]. However, the benefit of bALP lies in the fact that this isoenzyme is a direct product of bone metabolism, while PTH as a biomarker reflects parathyroid activity and is only an indirect indicator of bone metabolism. Also, the determination of bALP has several advantages from the analytical aspect: better stability, better analytical and intra-individual coefficient of variation [2].

Although the cut-off values for bALP in bone metabolism assessment depend on the method of determination, the initially recommended values of bALP specific electrophoretic technique were 27 U/L with 84% sensitivity and 70% specificity, with 90% positive predictability for high bone turnover and 58% positive predictability for low bone turnover [8] [9] [10] [11]. Cut-off value of 53 U/L had 100% specificity and 100% sensitivity, with 84% positive predictability for high bone turnover and 100% positive predictability for low bone turnover [8] [9] [10] [11].

The aim of this study was to compare bALP values determined by the electrophoresis technique and immunochemical method in patients on dialysis in order to examine the agreement between these two methods and the impact on the appropriate assesments of bone metabolism in these patients compared to the initially recommended limit values for bALP.

## Materials and Methods

### Patients

This study was conducted in 2015, and it included 71 patients (mean age: 56 ± 14.6, 44 years old, 44 males) undergoing maintenance hemodialysis (duration: 5 years and 17 months). All patients were treated at the Clinic of Nephrology, Clinical Center of Serbia. Prior to the study all the patients agreed to participate and gave their informed consent. Blood samples were drawn immediately prior to dialysis session and centrifugated within an hour. We constituted aliquots of samples and froze them at -70 °C until determination within the next two months. Practical work was conducted at the Laboratory for Medical Biochemistry Analysis, Faculty of Pharmacy, University of Belgrade and the Center of Medical Biochemistry, Clinical Center of Serbia. All patients gave informed consent to participate in the study, which was conducted according to the Helsinki Declaration and approved by the Ethics Committee of the Clinical Center of Serbia.

### Methods

We determined bALP activity using commercial kits for agarose gel electrophoresis technique (HYDRAGEL 7 and 15 ISO-PAL gels) on Hydrasis semi-automatic electrophoresis system (ISO-PAL, Sebia, France). Isoforms of total alkaline phosphatase were identified after sample migration in duplicate, using lectin for precipitation of bone isoenzime in one track, and quantified using specific chromogenic substrate. The value of bone isoenzyme is obtained by subtracting the densitometric values of liver-1 and placental-1 fractions obtained from the lectin track, from the value of the sum of liver-1, bone and placental-1 fraction of the »without« lectin track. All other fractions, liver-2, three separated intestinal fractions, placental-2 and the lipo complex are determined on the track without lectin. We used commercial quality control material, wich contains a human serum pool with alkaline phosphatase isoenzymes, as internal quality control (ISO-PAL Control (PN 4793), Sebia, France). Percentage on HYDRAGEL 7 and 15 ISO-PAL for liver-1+bone izoenzymes is 75.4% ± 7.8. Manufacturer’s recom - mendation is to include one track of ISO-PAL Control into each run on Hydragels (on CTL track) without any lectin solution. According to the Manufacturer’s performance specification analytical sensitivity is 2–3 U/L, dynamic range 50–600 U/L and within and between gels coeficient of variation is < 6.5% and < 4.1%, respectively. ISO-PAL electrophoresis technique was chosen as a comparative method.

bALP concentrations were also measured using commercial one-step immunoenzymatic Access OSTASE chemiluminiscence immunoassay (CLIA) (Access OSTASE, Beckman Coulter, Inc.). In this immunoassay, mouse monoclonal antibody specific to bALP was added to a reaction vessel with paramagnetic particles coated with goat anti-mouse polyclonal antibody and chemiluminiscence of the solid phase/capture antibody/bALP complex was measured after adding supstrate Lumi-Phos* 530. We used commercial quality control material (Access OSTASE QC, Beckman Coulter, Inc.), level 1 and 2 (human bALP at a level of approximately 29.3 and 120 U/L in matrix buffered bovine serum albumin with surfactant). We tested quality control specimens with each analytical run. According to the Manufacturer’s performance specification analytical sensitivity is 0.1 U/L, dynamic range 0.1–120 U/L and imprecision 4.8% and 5.3% for low and high internal quality control, respectively.

Reffering to a liver isoenzyme reactivity, Manufacturer suggests that 100 U/L of bALP activity yields a result of 37.5 U/L in the Access OSTASE assay. We used this conversion factor to uniform units of bALP measurement with two different methods and express all results as acivities (U/L).

### Statistics

We have checked normality of the distribution for the analyzed data using Kolmogorov-Smirnov test and for subgroups with data number less than 50 we used Shapiro-Wilkins test. We presented results as median and interquartile range (IQR). We used Passing-Bablock regression to assess the relationship between bALP values measured with two different assays. To additionally estimate agreement between two methods we have determined Concordance correlation coefficient for nonparametric data (Kendall's W). Bias in regression (Passing-Bablok analysis) and the Spearman correlation coefficient (Spearman's rho) was calculated to estimate relationship between continuous variables. The constant bias between analysed assays was evaluated from the intercept of the Passing-Bablok regression line, and the proportional bias was evaluated from the slope of the regression line. We have also determined residual standard deviation (RSD) ± 1.96 RSD interval as a marker of random differences, and conducted Cusum test to check linearity of the relationship between two data sets. In cases in which the Spearman's rho indicated poor correlation between two assays (R^2^ < 0.500), Kendall's W was not considered relevant to estimate agreement. If the correlation coefficient was substantial (R^2^ > 0.500), the Kendall's concordance correlation was evaluated. The linear correlation coefficient R provides a measure of linear relation between two sets of scores without specifying any degree of correspondence between the two sets of values. On the other hand, the Kendall's W provides a measure of reliability that is based on covariation and correspondence and has smaller gross error sensitivity (i.e. more robust) and a smaller asymptotic variance (i.e. more efficient). All statistical analysis were performed using MedCalc Software bvba (Version 2.5.0.0., Ostend, Belgium) and IBM SPSS Statistics version 21.0 (Chicago, IL).

## Results

**Figure 1:**
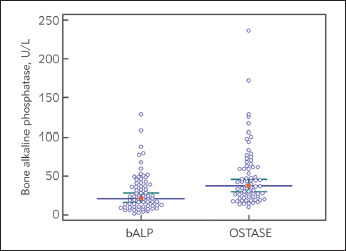
Distribution of bALP concentrations determined in 71 hemodialyzed patients regarding the used electrophoresis technique (bALP, U/L) and immunoassay method (OSTASE, U/L) The solid line represent median value and first quartile range

Using electrophoresis technique, we found that the median bALP concentration in all the analyzed patients was 21.2 U/L (IQR: 11.1-39.5 U/L) and ranged from 1.7-128.3 U/L. Using Access OSTASE method, the converted median bALP concentration into the same units as ISO-PAL was 37.4 U/L (IQR: 23.9-62.3 U/L) and ranged from 10.1-236.3 U/L. The distribution of the results according to the used method is presented in Figure 1. Using electrophoresis technique we have detected presence of the intestinal isoenzymes of alkaline phosphatase in 55% of patients with median value of 30% (IQR: 21.7-43.1%) (Manufacturer recommendation for cut-off for these izoenzymes is < 14%). In addition, we have detected presence of liver-2 alkaline phosphatase isoform in 42% of patients with median value of 16.6% (IQR: 11.8-22.4%) (Manufacturer recommendation for cut-off for this isoform is < 9%).

**Figure 2:**
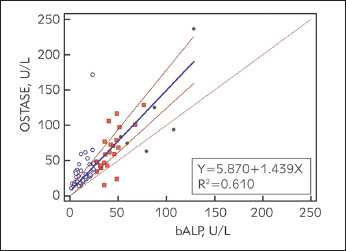
Passing-Bablock graphic present linear relationship between the two different methods used to determine concentrations of bone isoenzyme of alkaline phosphatase (electrophoresis technique, bALP, U/L and immunoassay method, OSTASE, U/L). Three different dot shapes and colours present three subgroups of measured isoenzyme concentrations according to the specified cut-off values of < 27 U/L, 27–53 U/L and ≥ 53 U/L for low, normal and high bone turnover, respectively

**Figure 3:**
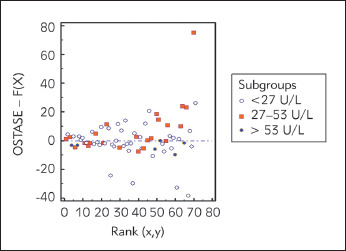
Passing-Bablok regression residuals plot present random differences between two analyzed methods regarding the three subgroups according to the specified cut-off values for low, normal and high bone turnover

Passing-Bablock regression is presented in Figure 2. with specified equation of the regression line between the analyzed methods: y=5.87+1.44x (R^2^ =0.610). 95% confidence interval (CI) for systematic differences was -0.64-10.39, for proportional differences was 1.25-1.68, and for random differences ± 1.96 RSD interval was -28.24-28.24. We presented Passing-Bablok regression residuals plot in Figure 3. that is a measure of random differences between the analyzed methods, with RSD of 14.41. The analyzed methods showed significant correlation (Spearman's rho: 0.776, P<0.01), but we found statistically significant (P<0.01) positive bias (27%) for the results measured by Access OSTASE compared with those obtained with ISO-PAL. The Kendall's W of 0.787 (P<0.0001) revealed significant concordance between two analysed methods. Cusum test showed no significant deviation from linearity (P=0.850).

According to the ISO-PAL, 59.2%, 30.9% and 9.9% of the patients presented bALP concentrations ≤ 27 U/L, between 27 and 53 U/L, and 53 U/L, respectively. Using the regression equation, we calculated equivalent concentrations obtained with two methods, when the value measured with the ISO-PAL is 27 and 53 U/L, that we presented in the Table 1. In addition, in the Table 1 we presented Spearman’s rho and Kendall’s W between ISO-PAL and Access OSTASE to classify the patients accordingly.

**Table 1:** Equivalent concentrations obtained with Access Ostase, when the value measured with the ISO-PAL is 27 U/L and 53 U/L, Spearman’s rho coefficient of correlation and Kendall's W coefficient of concordance between the analyzed methods to classify identically the patients when they present values ≤ 27 U/L, between 27 and 53 U/L, and ≥ 53 U/L with the ISO-PAL

ISO-PAL bALP concentrations	Equivalent Access Ostase bALP concentrations	Spearman’s rho (two-tailed probability)	Kendall’s W (two-tailed probability)
27 U/L (N=42)	44.75 U/L	0.598 (P < 0.001)	1.000 (P < 0.001)
27–53 U/L (N=22)	44.75–82.19 U/L	0.616 (P = 0.002)	0.669 (P < 0.001)
53 U/L (N=7)	82.19 U/L	0.429 (P = 0.337)	N/A

## Discussion

Electrophoresis technique is used as a screening method to assess relative part of bone isoenzyme regarding the total alkaline phosphatase activity, while using the immunochemistry assay it is possible to measure the exact bone isoenzyme concentration. Although these two methods have different measuring principles, the obtained comparison parameters in this study present good agreement between two independent methods for bALP determination, after we have conversed measured concentrations to the same units. Although, so far, there are not many published studies analyzing the comparison of the electrophoretic and immunochemical method of bALP determination, the literature data generally indicate an inappropriate agreement between the methods [2] [4] [6]. In addition, data analyses and interpretation is complicated issue that has been discussed for decades and still there is no gold standard for statistical procedure that should be used for method comparison data analyses [12]. Also, there are various interpretations of the matching coefficients and statistical tests in general. In addition to the different measurement principles of two analyzed methods, disagreement can be a result of different units of activity expression, i.e. concentrations of bALP. So far, there are no generally accepted recommendations for the conversion of activity units into bALP units, which makes the values of this isoenzyme significantly different between these two methods. However, disagreement most likely lies in the significant interference of other alkaline phosphatase isoenzymes that impede with the accurate and precise determination of the bALP using the immunochemical method, as well as the specificity of the detection antibodies. The obtained data in our study, indicate a significant presence of intestinal isoforms and liver-2 isoenzymes, which most likely contribute to higher values of detected bALP by the immunochemical method compared to electrophoretic technique. This support findings of Zhan et al. who pointed out that the accuracy of electrophoresis was comparable to that of immunochemistry assay when percent of serum bALP levels were high in patients with liver disease, but that accuracy of electrophoresis, further needs to be assessed, with patient samples under certain disease and conditions [13].

Opposite to the study of Ahmed et al. [4], our results indicate a significant positive bias of 27% for concentrations measured by the immunochemical method, which can be explained by the presence of isoforms of other isoenzymes of total alkaline phosphatase, that have been detected as bALP in immunoassay. In cited study, analyzed patients had Paget's disease, and there was no significant agreement between the electrophoretic technique and the immunoassay method. They found negative bias of immunoassay concentrations, the effect of interference was not shown, and the number of analyzed patients was small (N = 12). However, the authors have noticed that suspected results can occur only at very high concentrations of bALP (greater than 140 U/L), in which case one should be careful in interpreting the results. More than a decade ago, a group of Slovenian authors analyzed the association of activity and mass concentration of bALP measured with immunoassay in pre and postmenopausal women [14]. The results of their study showed that the activity and mass concentration of bALP significantly correlated, both with each other and with bone density parameters. However, in patients on dialysis, the presence of uremic toxins and additional bALP isoforms interfere with the adequate activity of bALP, so interpretation of the results may be problematic, especially in the case of high con centrations of bALP, when measured using immunoassay [15] [16].

The obtained CI of 95% of intercept and slope in our study, using Passing-Bablock regression analysis, indicate that there is no random and systematic differences between the analyzed methods, but revealed proportional differences in measurement. This suggests that considerable efforts should be performed to standardize bALP assays. Besides, using analysis of residuals, as difference of the observed OSTASE concentration with the value predicted by the regression equation for the corresponding bALP concentration, we found the remaining variation after correcting for systematic and proportional differences was minimal, as 95% of the residuals lie in the interval ± 1.96 times the RSD. Although, Passing Bablock residual plot revealed good agreement for subgroups of patients with bALP > 53 U/L, concordance coefficient for this subgroup was not applicable, most likely because of the small number of patients (N=9). The other two analyzed subgroups (≤ 27 U/L and 27-53 U/L) represented significant correlation and concordance coefficient. However, Yessayan et al. [17] found, that compared to iPTH, bALP was shown to be the optimal predictor of biopsy findings with higher concentrations and an optimal cut-off at 58 U/L.

Limitation of this study is that we arbitrary accepted electrophoresis method as referent method, since this is originally method we routinely use in our laboratory. We used initially recommended cut-off values for bALP for low, normal and high bone turnover. We are aware that these values are debatable and that they may not be appropriate for nowdays methods. In addition, we had insufficient number of patients in subgroup with higher bALP concentrations, although Passing Bablock residual plot revealed good agreement for this subgroup.

We can conclude, based on our results, that immunoassay displays significant positive bias, in relation to electrophoresis, in the presence of other alkaline phosphatase isoenzyme. Clinicians should be aware of this phenomenon when they interpret bALP results. It is important to conduct future studies that include bone biopsy data, to determine adequate clinical decision limits, for appropriate clinical utility of bALP as a direct marker of bone metabolism. Although immunoassays are increasingly used, as fully automated methods, in a large number of laboratories and become routine methods for bALP determination, it should be beared in mind, besides various interferences, also the heterogeneity of the bALP itself, especially in HD patients.

*Acknowledgment*: This study was supported by the Ministry of Science of Serbia on the basis of contract No.175036.

### Conflict of interest statement

The authors state that they have no conflicts of interest regarding the publication of this article.

## List of abbreviations

bALP, bone alkaline phosphatase; HD,hemodialysis; PTH, parathyroid hormone; IQR, interquartilerange; CI, confidence interval; RSD, residual standarddeviation.
